# A Novel Treatment of Opioid Cravings With an Effect Size of .73 for Unilateral Transcranial Photobiomodulation Over Sham

**DOI:** 10.3389/fpsyt.2020.00827

**Published:** 2020-08-19

**Authors:** Fredric Schiffer, William Reichmann, Edward Flynn, Michael R. Hamblin, Hannah McCormack

**Affiliations:** ^1^ MindLight, LLC, Newton Highlands, MA, United States; ^2^ Developmental Biopsychiatry Research Program, Mclean Hospital and Department of Psychiatry, Harvard Medical School, Belmont, MA, United States; ^3^ Independent Consultant, Danvers, MA, United States; ^4^ Wellman Center for Photomedicine, Massachusetts General Hospital, Harvard Medical School, Boston, MA, United States; ^5^ Laser Research Centre, Faculty of Health Science, University of Johannesburg, Doornfontein, South Africa

**Keywords:** opioid craving, depression, anxiety, photobiomodulation, brain hemispheres, laterality, dual-brain psychology

## Abstract

**Background:**

Opioid use disorders (OUDs) are an epidemic causing catastrophic consequences to individuals, families, and society despite treatments including psychotherapy, substitution therapy or receptor blockers, and psychoeducation. We have developed a novel treatment that combines unilateral transcranial photobiomodulation (t-PBM) to the hemisphere with a more positive valence by Dual Brain Psychology (DBP).

**Methods:**

We used a randomized, double blind, placebo-controlled protocol in which 22 patients with significant opioid cravings and a history of recent or current OUD attended three 1-h weekly sessions. After baseline measures of opioid craving and other psychometrics, subjects received two unilateral t-PBM applications (810 nm CW LED, 250 mW/cm^2^, 60 J/cm^2^, 4 min) or a sham (foil-covered LED) at F3 or F4. Prior to any treatment we used two tests to determine which hemisphere was more associated with a negative outlook and cravings and treated that side before the more positive hemisphere. Primary outcome measure was an opioid craving scale (OCS). Secondary outcomes were weekly Hamilton Depression (HDRS) and Anxiety (HARS) Rating Scales prior to treatments and at follow-up.

**Results:**

Immediately after treatment the OCS improved significantly for both the sham and active treatments, but one week later the active treatment showed a 51.0% (SD 33.7) decrease in OCS while a week after the sham treatments there was a decrease of only 15.8% (SD 35.0) (by Wilcoxon Sign Rank Test, p = 0.004) and by a mixed model it was p = 0.0071. The effect size for the differences between active and sham was 0.73. For the active treatment from before and after treatment the effect size was 1.51 and for the sham, 0.45. The HDRS improved from a baseline of 15.1 to 8.8 (SD 10.3) a week after the active treatment and to 13.3 (SD 12.9) after the sham (p = 0.0071). HARS improved from 14.7 to 8.0 (SD 13.2) after the active treatments and to 14.3 (SD 16.0) after the sham, p = 0.08. Active treatment of the positive hemisphere after the negative hemisphere significantly improved the OCS, but there was no significant difference after the sham treatment. One patient complained of 2 h of abdominal bloating and dropped out; no other adverse effects were observed.

**Discussion:**

Unilateral t-PBM to the hemisphere with a more positive hemispheric emotional valence was an effective and safe treatment for opioid cravings as well as for depression and anxiety. Our results also lend support to the underlying premises of DBP.

## Introduction

According to the National Institute on Drug Abuse, “The combined healthcare, crime-related, and productivity costs of tobacco, alcohol, and illicit drugs exceed $700 billion a year, but dollars only poorly approximate the devastating human cost of substance use disorders” (1). In the US in 2015, there were over 52,000 drug overdose deaths, an increase of 11% over 2014 (2–4). Drug abusing patients demonstrate mental distress, health complications, loss of productivity, and increased criminality, which are injurious not only to patients and their families, but also to society as a whole. We have obtained preliminary data (5) that a simple, painless, inexpensive, and safe treatment called unilateral transcranial photobiomodulation (tPBM) using near-infrared light (NIR) may effectively benefit patients with a history of opiate dependence by improving their psychological well-being.

The physiological benefits to the brain from tPBM have been the subject of a rich literature of >400 papers on PubMed and several reviews (6–10). The reviews describe in detail how NIR light is absorbed by cytochrome-C oxidase, which stimulates ATP formation in the mitochondria (11). tPBM also increases neurotrophic factors in the brain, increases blood flow, and decreases inflammation (8, 12).

Hemispheric emotional valence has generally been considered in three hypotheses. The first is the right brain hypothesis, which posits that the right hemisphere is specialized for all emotional responses (13, 14). The second is the motivational hypothesis (15, 16), which suggests that approach emotions (including anger) are associated with the left hemisphere and that withdraw emotions are associated with the right. The third hypothesis, the valence hypothesis (17, 18), proposes that the left hemisphere is associated with positive emotions and the right with negative emotions.

Schiffer originally proposed Dual-brain Psychology (DBP) in 1997 (19–22), which posits that one brain hemisphere tends to be relatively mature and healthy, while the other hemisphere may be more affected by past traumas and supports a personality that is more prone to immature and/or destructive beliefs and/or behaviors. Schiffer has been concerned not simply with positive or negative emotions, but with entire personalities that differ in that one associated with one hemisphere tends to be associated with a disposition that is more mature and grounded in present reality and that the other, associated with the opposite hemisphere, seems to be more affected by past traumas, especially childhood traumas, and has a more childlike disposition that is more strongly influenced by past adversities. His hypothesis came out of his clinical observations, his rereading of the split-brain studies (23), studies by Wittling (24), and a number of published experiments by him and his associates on split-brain patients (25), fMRI imaging during lateral visual field stimulation (26), probe auditory evoked potentials during emotional memories (27), the use of HEV to successfully predict rTMS outcome in two studies on depression (28, 29), and a study using tPBM to treat anxiety and depression (5). The present study combined the physiological benefits of tPBM with the use of Schiffer’s HEV to guide the treatment to the positive hemisphere.

**Figure 1:**
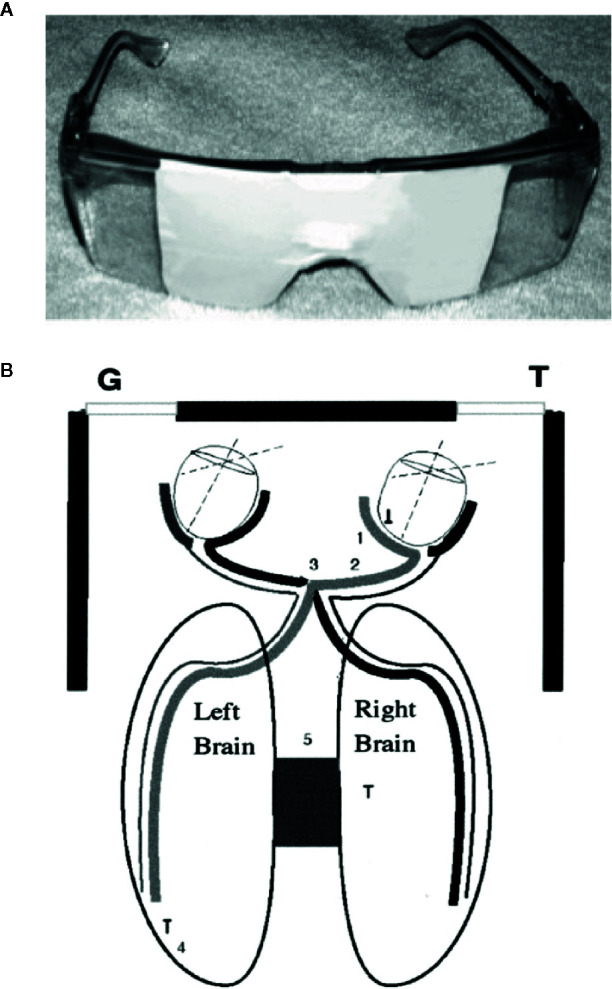
**(A)** Taped safety glasses used in the experiment. **(B)** A diagram of how looking to the left allowed vision primarily out of the LVF and looking to the right allowed vision primarily out of the RVF. (1) Nasal retina; (2) optic nerve; (3) optic chiasm; and (4) occipital cortex. The diagram shows how the medial retina receives light from the lateral visual field and transfers information to the contralateral hemisphere. These neurological facts were used extensively in the split-brain studies, and we assume that the personality changes described here and in our other publications use a similar mechanism (26).

Contrary to the established models that have generally associated negative emotions with the right hemisphere, several findings from WADA studies (30, 31), metanalyses of functional imaging studies (32, 33), and studies of post-stroke patients (34–36) indicated that negative emotions can often be left hemisphere dominant. Schiffer has reported that the side that is less symptomatic, which thereby has a positive HEV varies between individuals, but strongly tends to be a trait for any individual. Schiffer’s HEV is determined simply by asking the patient to look out of either the left or the right lateral visual field by blocking his vision with safety glasses taped to allow vision out of one visual field and reporting his feelings and thoughts. He might be asked to look at a provocative photograph of an angry person while looking out that visual field, based in part on the pioneering work by Wittling and associates (24). The patient might be asked to rate this level of depression from 0 to 10. Then he is asked to look out the other lateral visual field and repeat the requests. In about 70% of patients there is at least a 2-point difference between the sides. Further, the patient is apt to see the therapist as critical (perhaps, as his mother was), to dislike himself, and to develop drug or gambling cravings while looking out of the negative side, all of which are reversed out of the other visual field. The visual field that is less symptomatic relates to the contralateral hemisphere which is then considered positive. See **Figure 1**. Recently, we have developed a computer test for HEV that correlates with lateralized volumes of the n. accumbens and amygdala. In two studies of rTMS, those patients who by this simple visual test had a positive left hemisphere had a far superior outcome compared to those with a negative left hemisphere from a course of two weeks rTMS, which only by tradition is only given to the left side of the head (28, 29).

Schiffer found that in his practice using unilateral tPBM to the positive hemisphere at either F3 or F4 gave very superior results compared with bilateral tPBM at F3 and F4 (5). See **Table 1**.

**Table 1:** A comparison of bilateral tPBM and unilateral tPBM.

Bilateral tPBM	Unilateral tPBM
No obvious improvement; requires HDRS to observe improvements	Immediately after Rx report 84% of patients report profound or moderate improvements which on average last for about 3 days
Modest improvements 2 weeks after of Rx	Immediate observable improvement
No long-term clinical experience, except for one case report	Two years of experience in clinical practice with opiate disorders with more than 1,000 treatments given as an off-label, adjunctive treatment
No serious side-effects observed	No serious side-effects observed
Not related to any psychological theory	Integrated with Dual-Brain Psychology

## Aims of The Study

Firstly, to test whether unilateral tPBM to stimulate the positive hemisphere in patients with a current or recent history of opioid use disorders (OUD) could significantly reduce opioid cravings in a randomized, double blind, placebo-controlled design.

Secondly, to evaluate the effects of unilateral tPBM on affect as measured by the Hamilton Depression Rating Scale (HDRS) and the Hamilton Anxiety Rating Scale (HARS).

Thirdly, to test the hypothesis that treating the positive hemisphere is superior to treating the hemisphere with a more negative HEV using active treatment. Applying the sham treatment to the positive hemisphere should not be superior to applying it to the negative hemisphere. We wanted this aim to further test Schiffer’s DBP hypothesis. Schiffer had found clinically that applying tPBM to the negative hemisphere before the positive resulted in the patient’s still having the positive benefit of the treatment of the positive hemisphere.

Our fourth aim was to confirm that there were no significant side effects of tPBM as found in other reports (5, 6, 8–10, 37, 38).

## Materials and Methods

This single site, double blind, placebo-controlled study was approved by the New England IRB, Needham, MA. Twenty-two patients who reported active opioid cravings and had a current or recent opioid use disorder were recruited from online advertising between July and September 2019. Each went through the consent procedure and gave written consent for the study, following which they were given a clinical medical and psychiatric history.

### Inclusion and Exclusion Criteria

Inclusion criteria were that the patients complained of opioid cravings and were between the ages of 18 and 65. Each met the criteria for a history of opioid dependence by DSM V. Patients who were receiving treatment for opioid dependence or other psychiatric disorders, either psychological or pharmacological treatments, could continue their treatment during the time of the study but were asked to try not to alter their treatment from the onset of the experiment until its conclusion. No patient requested to alter his regular treatment. Subjects were recruited without regard to gender or ethnicity and were enrolled on a first come basis. Exclusion criteria were past history of psychotic disorder (including schizophrenia or schizoaffective disorder), a history of violent behavior, a history of a past suicide attempt, a history of current suicidal ideation, a history of a neurological condition (*e.g.* epilepsy, traumatic brain injury, stroke), pregnancy, any current acute or chronic medical condition that might confound the study. Any patient judged by an investigator to have an impaired decision-making capacity would have been excluded. In actuality, no patient seeking recruitment was excluded after a screening phone call or clinical interview.

### Study Design and Treatment

The patients attended the laboratory for three consecutive weekly visits. Patients were not allowed to reschedule a missed session so that the timings of the treatments would be consistent. At the first visit the experimenters gave the patients two tests for HEV to determine the positive hemisphere. The two tests for HEV are the lateral visual field test (LVFT) and a novel computer test for HEV (CTHEV), which has been submitted for a US Patent (patent application number: 16703937). The LVFT consists of asking each patient to put on taped safety goggles that allowed vision out of only one visual field, when the patient looked as far as possible to the left or the right (5, 26, 27). See **Figure 1**. While looking out of each visual field the patient was asked to rate from 0 to 10 his level of distress and his level of opioid cravings while he looked at a photograph of a very angry man, designed so that each half of the face was identical. The CTHEV, showed a video of alternating, symmetrical photographs of angry men to one visual field (by having the patient fixate on a central dot for 1 min) after which he was asked to rate from 0 to 10 his level of distress and his level of opioid cravings. As with the LVFT, the CTHEV was then repeated to the other visual field and the side with the lower scores was considered the positive visual field, which suggested that the contralateral hemisphere was the positive hemisphere. The HEV with either test was the numeric difference between the recorded scores from each visual field.

At each of the three weekly visits before treatment each patient was also given a urine drug screen, and if female, a pregnancy test, as well as each of the baseline outcome measures, which were the Hamilton Depression Rating Scale-17 (HDRS) (39), the Hamilton Anxiety Rating Scale (HARS) (40), and the Opioid Craving Scale (OCS) (41). The OCS was our primary endpoint and was given before and after each treatment and the follow-up session. It consisted of a mean score from three questions each rated from 0 to 9. (1) Please rate how strong your desire for an opiate is right now. (2) Please rate how strong your urges would be for an opiate if something in the environment had reminded you (examples; seeing a spoon), a needle, a mirror, or an alcohol advertisement), and (3) Please imagine yourself in the environment in which you previously used drugs and/or alcohol (a bar, your dealer’s house, a shooting gallery, or whatever situation reminds you most strongly of active drug use). If you were in this environment right now, what is the likelihood that you would use an opiate?

At the beginning of each of the three weekly sessions, we obtained a detailed verbal report from the patient of his daily use of opioids over the previous week. We used this data (which we felt was honestly and accurately reported and was consistent with the urine drug screen) to calculate a timeline follow-back score using a typical unit of opioid use for the patient multiplied by the number of units and days of use.

**Figure 2:**
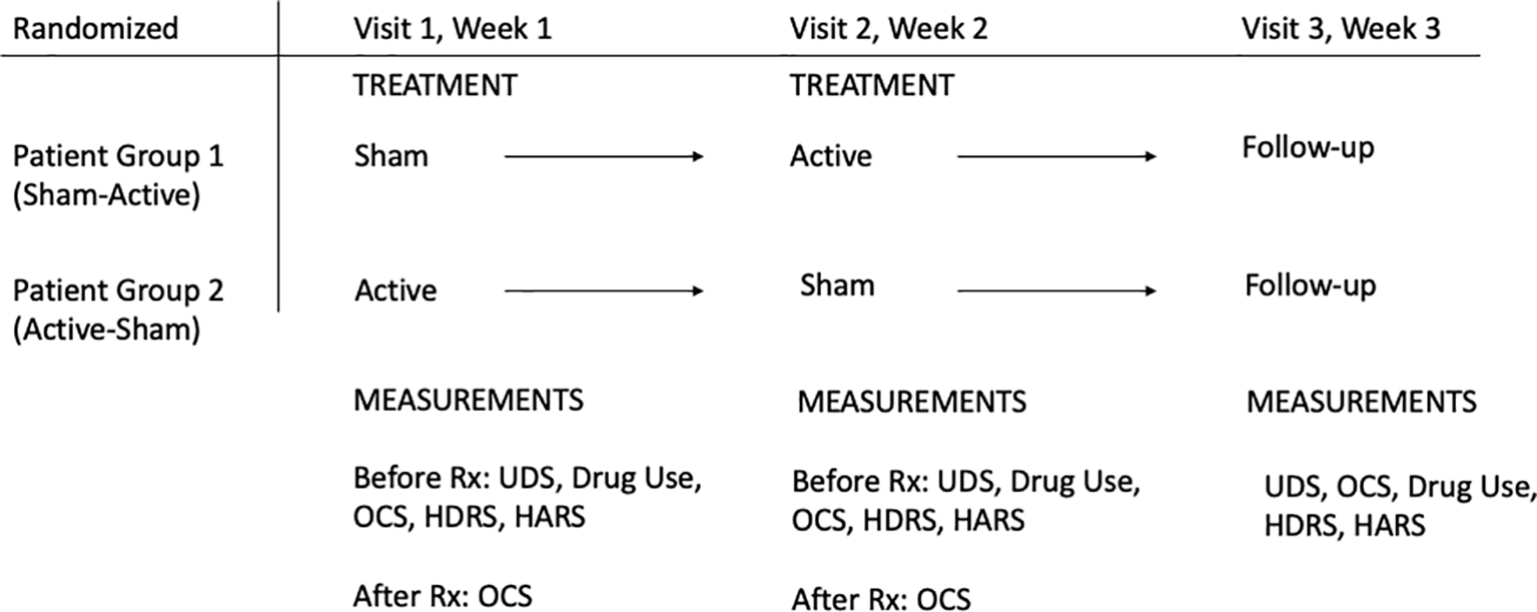
A diagram of the randomization and timing of the study treatments and measurements.

After the baseline measures each patient then was given either a sham treatment or an active treatment (unilateral tPBM) in subsequent weeks on a randomized basis. That is, on the first visit the patient was treated with either an active device or a sham device. On his second visit, he was treated with the opposite device. On the third weekly visit, he received only a follow-up evaluation. See **Figure 2**. Before and after each treatment (first to the negative hemisphere and then to the positive hemisphere), the patient was administered the OCS. At the follow-up session we gave the patients the OCS, UDS, HDRS, HARS, and a report of opioid use during the prior week. Using preliminary data from his private practice FS found that treating the positive hemisphere after the negative relieved any negative effects that the first treatment may have caused and left the patient in a more positive psychological state, while allowing a comparison of the effects of treatments to the negative and the positive hemispheres under active and sham conditions. See **Figure 2**.

### Randomization and Blinding

All treatments were randomized by a research assistant (EF) who also administered all the patient treatments, which were blinded to the two data recorders (HM) and (FS). The randomization procedure used computer generated random numbers blocked so that 60% would be given the sham condition before the active to try to avoid crossover effects.

### Device

We used the same light source that was used in our 2009 study (6). The active treatment consisted of applying tPBM from a light emitting diode (LED) array (Marubeni America Corp, Santa Clara, CA) with a peak wavelength of 810 nm (+40 nm), delivering 250 mW/cm^2^ when applied to the skin. tPBM for 4 min (total delivered fluence per site of 60 J/cm^2^) at each of two sites on the forehead that correspond to the 10–20 EEG sites, F3, and F4. Based on a penetration of 3.7% of the light to the dura, we calculated that 2.1 J/cm^2^ was delivered to each of the treated areas of the brain. The level of light exposure at the skin was well below the irradiance allowed by the ANSI standard of 320 mW/cm^2^. The New England IRB currently and the Partners IRB in 2009 both determined that the device posed no significant risk. In other published tPBM studies no significant side effects have been reported to date (5, 8, 9, 12, 37, 42–47).

The LED was attached to a heatsink and a fan for cooling. If the patient wanted further cooling, he could tilt the device so that the LED was a few millimeters from the skin and allowed more air flow from the fan. The power supply was constructed by a product engineer so that the LED would deliver 250 mW/cm^2^ at the skin. The sham device was the same device, except that the LED was covered with aluminum foil so that the patient felt the same warmth but received no light as verified by a photon detector. In this and other trials, subjects could not detect whether they were receiving active or sham treatment other than by their psychological response.

### Analysis

A biostatistician (WR) selected and conducted all of our statistical tests using R (48). Our main statistical tests were mixed model analyses and Wilcoxon Sign Rank Tests, depending on the statistical need. Because our hypothesis was that the active condition would obtain better clinical results, we used one-sided tests. A copy of all study data and of the statistical tests performed and their results are contained in the **Supplementary Material (SM)**. In the *Results* section, we present the results extracted from the SM that we feel most accurately present our findings without repetition or data overload. In the SM, we noted which tables were included in the main paper as well as their table numbers which were different from the numbers in the SM.

## Results

### Baseline Characteristics

We initially enrolled 22 patients whose overall baseline characteristics are described in **Table 2** and a more complete description is in the SM. We categorized those patients who received the active treatment the first week and the sham the second week as Active–Sham and the other group as Sham–Active. We attempted to enroll female subjects but were only able to enroll two, one of whom dropped out after the two treatments but before the follow-up week because she got a job with a conflicting schedule. For males, one patient dropped out before the follow-up session. Three others, who were all in the Active–Sham group, dropped out after the first treatment. Of the five patients who dropped out, four gave reasons that we felt, and the patient reported, were unrelated to the study. For example, one patient reported that he dropped out because he “had to get out of town.” Only one patient dropped out because of a possible adverse reaction. He had what was described in a phone conversation, initiated by (FS), as “bloating in his abdomen,” which resolved quickly with self-treatment with Pepto-Bismol the evening of the study.

**Table 2:** Baseline demographic and clinical characteristics.

Characteristic	Active–Sham (n = 9)	Sham–Active (n = 13)	All Patients (n = 22)
Age Mean (SD)	52.6 (9.8)	46.5 (9.2)	49.0 (9.7)
			
Gender			
Male	8 (88.9%)	12 (92.3%)	20 (90.9%)
Female	1 (11.1%)	1 (7.7%)	2 (9.1%)
			
Race/Ethnicity			
Black	6 (66.7%)	5 (45.4%)	11 (50.0%)
White	3 (27.3%)	8 (72.7%)	11 (50.0%)
			
Handedness			
Left	2 (22.2%)	1 (7.7%)	3 (13.6%)
Right	7 (77.8%)	12 (92.3%)	19 (86.4%)

All Randomized Patients with Complete Data.

### Immediate Results From Treatment

Immediately after the active and the sham treatments we found large and significant decreases in the OCS in both groups. Active had a mean (SD) percent decrease from immediately before treatment of 44.1% (39.6), and the sham had a decrease of 45.5% (35.6), both of which had a p value < 0.0001, by a one-sided Wilcoxon Test. The difference between conditions was not significant, p = 0.445. However, we found that when we compared the percent decrease in OCS one week after the treatments, there were significant differences between the active and the sham treatments.

### One Week Post-Treatment Results

**Figure 3:**
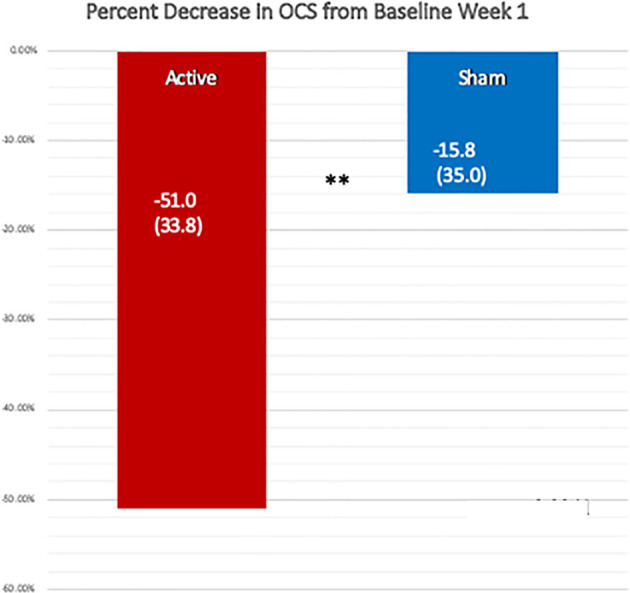
Comparison of the percent reduction in the Opioid Craving Scale one week after the active and the sham treatments, using the OCS from week 1 before treatment as the baseline. The change after the active treatment, −51.0% (SD 33.7) was significantly greater than that following the sham treatment, −15.8% (SD 35.0), by a one-sided, Wilcoxon Test, **p = 0.004. Cohen’s ∂ was 0.73 for the active treatment over sham.

When we compared the active and sham conditions for all remaining 17 patients we found, as shown in **Table 3** and **Figure 3**, a percent decrease in OCS of 51.0% (33.7) for the week following the active treatment and only 15.8% (35.0) for the week following the sham, p = 0.0040, one-sided Wilcoxon test. For this analysis we chose using the week 1 baseline because the values were common for all patients and were measured before receiving any study intervention. More patients (71%) received the active treatment in week 2, because we wanted to avoid follow through effects with a blocked randomization. For the active over the sham the effect size, Cohen’s ∂, was.73. For the active treatments from their baseline the Cohen’s ∂ was 1.51, while for the sham treatments it was 0.45.

**Table 3:** Comparison of mean OCS scores (percent change) between active and sham treatments one week after treatment.

Statistic	Baseline (Week 1)	Active	Pct Difference (Active − Baseline)	Sham	Pct Difference (Sham − Baseline)	Difference of Pct Differences (Active − Sham)
N	17	17	17	17	17	17
Mean (SD)	6.9 (1.3)	3.5 (2.7)	−51.0 (33.7)	5.8 (2.6)	−15.8 (35.0)	−35.2 (48.2)
Median	7.0	3.3	−60.0	6.7	−4.2	−31.8
Min, Max	4.3, 9.0	0.0, 8.3	−100, 0.0	2.0, 9.0	−72.7, 33.3	−115, 52.4
95% CI	(6.2, 7.6)	(2.1, 4.9)	(−68.3, −33.7)	(4.5, 7.2)	(−33.7, 2.2)	(−60.0, −10.5)
p-value (paired t-test)			<.0001		0.0408	0.0041
p-value (signed-rank test)			<.0001		0.0898	0.0040

All Randomized Patients with Complete Data.

[1] Wilcoxon signed-rank tests were performed to assess percent change from baseline in the OCS score one week after active treatment and sham treatment, separately.

[2] A Wilcoxon signed-rank test was performed to compare the percent change from baseline in the OCS score on active treatment versus sham treatment (Difference of Percent Differences column). A positive value for the difference of percent difference favors sham treatment, while a negative value favors active treatment.

[3] One-sided lower p-values were calculated for all tests.

Nine of 17 (53%) in the active group had at least a 60% improvement in the OCS, using the week 1 baseline. Two additional patients who did not have a sham treatment also had greater than a 60% improvement, 11/19 (58%). In the sham group, including one patient who did not have an active treatment, only three of 18 (17%) achieved at least a 60% reduction in the OCS. As shown in **Table 4**, by McNemar’s test, a paired, one-sided test of N = 17, we compared the paired percent differences and found that it was significant, p = 0.029.

**Table 4:** Comparison of Clinically Meaningful Improvement in Mean OCS Between Active and Sham Treatments One Week After Treatment.

_	Active (n = 17)	Sham (n = 17)	P-value
Patients with decrease in OCS of at least 60% from baseline, n (%)	9 (52.9%)	3 (17.6%)	0.0289

All Randomized Patients with Complete Data.

[1] Clinically meaningful improvement in mean OCS was defined as a percent decrease from baseline (pre-treatment week 1) of at least 60%.

[2] McNemar’s test was performed to compare paired percentages and one-sided p-value was estimated.

**Table 5A** shows the results of a mixed model analysis of the percent change in mean OCS prior to treatment each week that included treatment, treatment sequence and week as fixed effects and subject level as a random effect. The active treatment *versus* the sham treatment had a one-sided p value of 0.0078. **Table 5B** shows that the active treatment had an adjusted mean of −51.93% (95% CI −69.21, 34.64), and the sham treatment had an adjusted mean of −22.17% (95% CI −41.22, −3.12).

**Table 5A:** Summary of Mixed Model for Percent Change in Mean OCS.

Parameter	Beta Estimate (Std Err)	95% CI	P-value
Intercept	−35.14 (11.74)	−59.80, −10.48	0.0078
Active–Sham Sequence (*vs* Sham–Active Sequence)	5.22 (13.12)	−22.74, 33.18	0.6962
Active Treatment (*vs* Sham Treatment)	−29.76 (10.92)	−53.04, −6.48	0.0078
Week 2 (*vs* Week 3)	20.73 (10.92)	−2.56, 44.01	0.0772

All Randomized Patients Non-Missing Data at Particular Visit.

Summary of Parameter (Beta) Estimates from Mixed Model.

[1] A mixed model for percent change in mean OCS assessed prior to treatment each week that includes treatment, treatment sequence, and week as fixed effects and a subject level random effect was constructed.

[2] Active Treatment (vs Sham Treatment) is a one-sided p value.

**Table 5B:** Summary of Mixed Model for Percent Change in Mean OCS.

Treatment	Adjusted Mean (95% CI)
Active	−51.93 (−69.21, −34.64)
Sham	−22.17 (−41.22, −3.12)

All Randomized Patients Non-Missing Data at Particular Visit.

Summary of Adjusted Means by Treatment from the Mixed Model.

[1] A mixed model for percent change in mean OCS assessed prior to treatment each week that includes treatment, treatment sequence, and week as fixed effects, and a subject level random effect was constructed.

**Figure 4:**
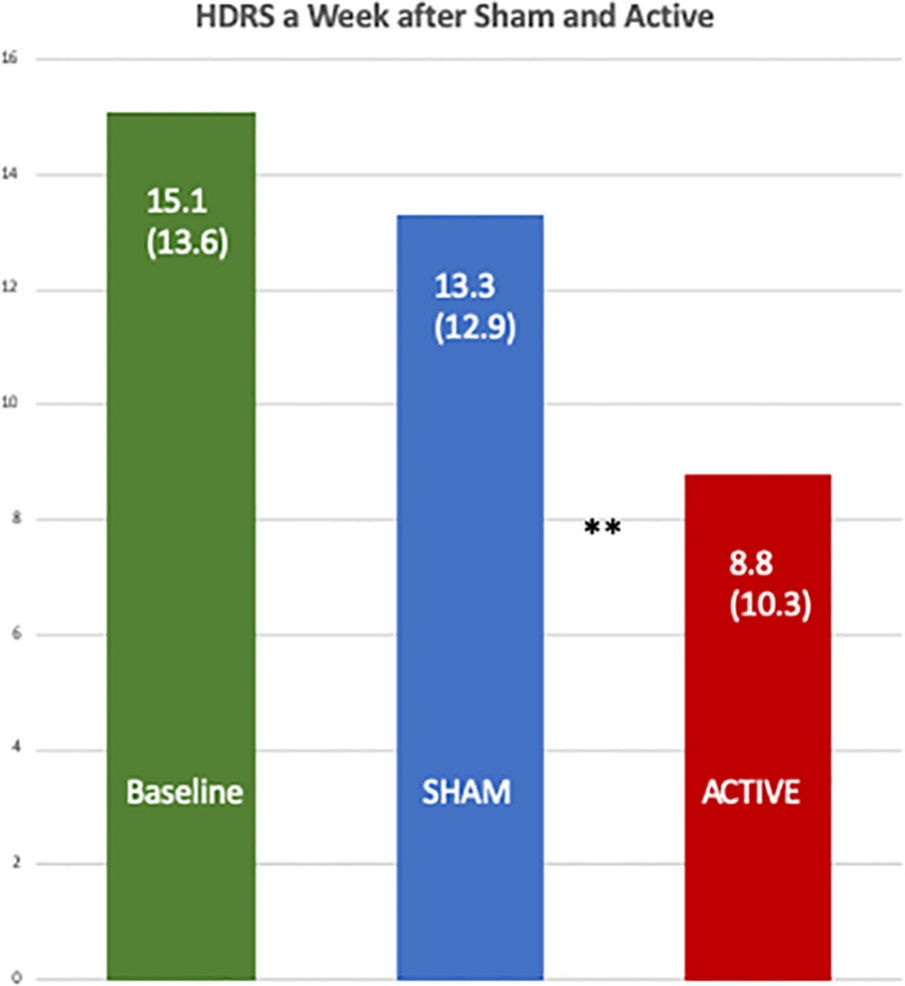
Comparison of the baseline Hamilton Depression Rating Scales with those one week after the active and the sham treatments. The differences between the scores after the active [8.8 (SD 10.3)] and sham treatments [13.3 (SD 12.9)] were significant by a one-sided, Wilcoxon Test, **p = 0.0053.

**Table 6** shows the data from a mixed model for the percent change in mean HDRS, and the active treatment had an adjusted mean of −7.97 (95% CI −57.50, 41.56), and the sham had 52.53 (95% CI −0.55, 105.60) with a one-sided p value = 0.0078. **Figure 4** shows a comparison of the raw HDRS scores at baseline and after the active and sham treatments and the p values from the one-sided Wilcoxon tests, including an active *versus* sham difference with a p value of 0.0053.

**Table 6A:** Summary of Mixed Model for Percent Change in Mean HDRS.

Parameter	Beta Estimate (Std Err)	95% CI	P-value
Intercept	16.54 (30.67)	−47.89, 80.97	0.5963
Active–Sham Sequence (*vs* Sham–Active Sequence)	120.73 (42.72)	29.11, 212.36	0.0135
Active Treatment (*vs* Sham Treatment)	−60.49 (21.59)	−106.81, −14.18	0.0071
Week 2 (*vs* Week 3)	−48.76 (21.59)	−95.08, −2.45	0.0404

All Randomized Patients Non-Missing Data at Particular Visit.

Summary of Parameter (Beta) Estimates from Mixed Model.

[1] A mixed model for percent change in mean HDRS assessed prior to treatment each week that includes treatment, treatment sequence, and week as fixed effects and a subject level random effect was constructed.

[2] Active Treatment (vs Sham Treatment) is a one-sided p value.

**Table 6B:** Summary of Mixed Model for Percent Change in Mean HDRS.

Treatment	Adjusted Mean (95% CI)
Active	−7.97 (−57.50, 41.56)
Sham	52.53 (−0.55, 105.60)

All Randomized Patients Non-Missing Data at Particular Visit.

Summary of Adjusted Means by Treatment from the Mixed Model.

[1] A mixed model for percent change in mean HDRS assessed prior to treatment each week that includes treatment, treatment sequence, and week as fixed effects and a subject level random effect was constructed.

**Figure 5:**
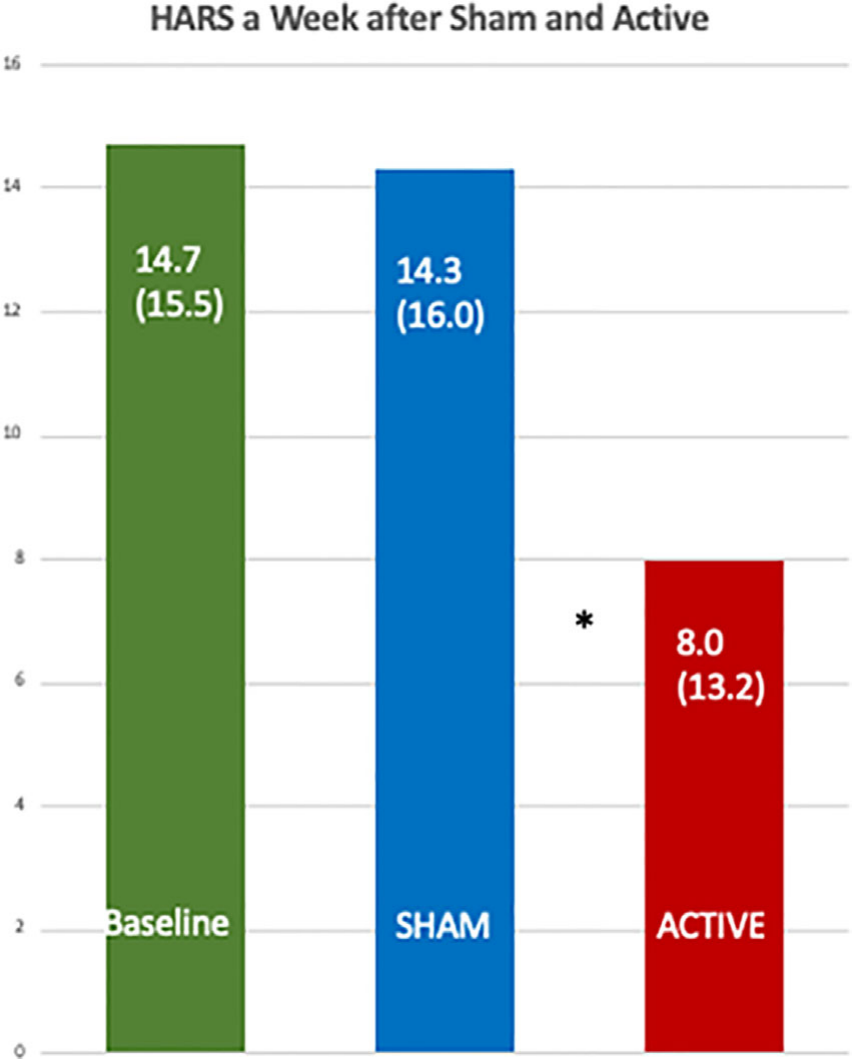
Comparison of the baseline Hamilton Anxiety Rating Scales with those one week after the active and the sham treatments. The differences between the scores after the active [8.0 (SD 13.2)] and sham treatments [14.3 (SD 16.0)] were significant by a one-sided, Wilcoxon Test, *p = 0.012.

**Table 7** shows the data from a mixed model for the percent change in mean HARS and the active treatment had an adjusted mean of 2.96 (95% CI −79.13, 85.06), and the sham had 53.96 (95% CI −33.54, 141.46) with a one-sided p value = 0.039. **Figure 5** shows a comparison of the raw HDRS scores at baseline and after the active and sham treatments and the p values from one-sided Wilcoxon tests, including an active *versus* sham difference with a p value of 0.0124.

**Table 7A:** Summary of Mixed Model for Percent Change in Mean HARS.

Parameter	Beta Estimate (Std Err)	95% CI	P-value
Intercept	41.46 (50.34)	−64.29, 147.22	0.4209
Active–Sham Sequence (*vs* Sham–Active Sequence)	51.37 (71.44)	−101.84, 204.59	0.4839
Active Treatment (*vs* Sham Treatment)	−51.00 (34.00)	−123.91, 21.92	0.0779
Week 2 (*vs* Week 3)	−26.39 (34.00)	−99.30, 46.53	0.4506

All Randomized Patients Non-Missing Data at Particular Visit.

Summary of Parameter (Beta) Estimates from Mixed Model.

[1] A mixed model for percent change in mean HARS assessed prior to treatment each week that includes treatment, treatment sequence, and week as fixed effects and a subject level random effect was constructed.

[2] Active Treatment (vs Sham Treatment) is a one-sided p value.

**Table 7B:** Summary of Mixed Model for Percent Change in Mean HARS.

Treatment	Adjusted Mean (95% CI)
Active	2.96 (−79.13, 85.06)
Sham	53.96 (−33.54, 141.46)

All Randomized Patients Non-Missing Data at Particular Visit.

Summary of Adjusted Means by Treatment from the Mixed Model.

[1] A mixed model for percent change in mean HARS assessed prior to treatment each week that includes treatment, treatment sequence, and week as fixed effects and a subject level random effect was constructed.

**Figure 6:**
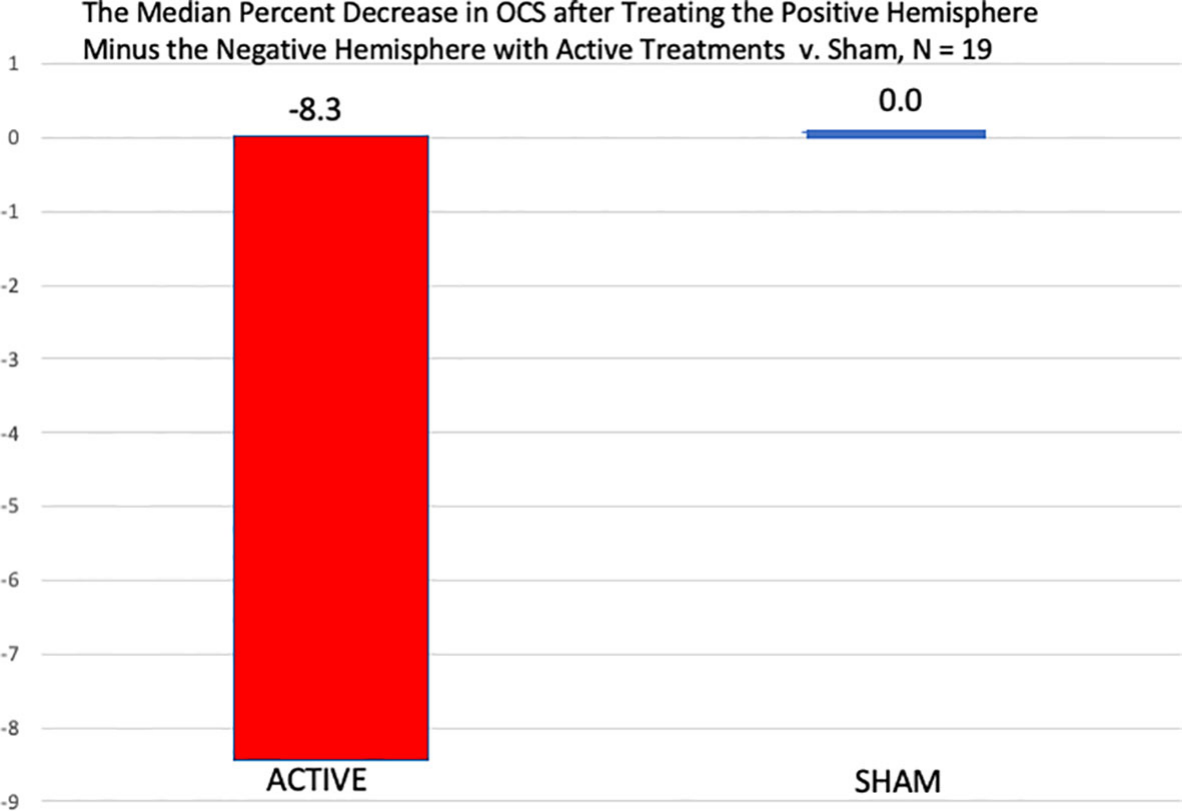
Comparison of the median percent reduction in the OCS measures immediately after the treatment of the negative hemisphere followed by the treatment of the positive hemisphere in both the active conditions and the sham conditions. In the active conditions there was a significantly greater percent decrease in OCS when the positive hemisphere was treated than the negative, by a one-sided, Wilcoxon Test, p = 0.021, but after the sham treatments, there was no difference between the two hemispheric sham treatments.

**Table 8** shows that when the more positive hemisphere (HEV by the LVFT and/or CTHEV tests) was treated during the active treatments, the OCS scores were lower (better) than when the hemisphere with the more negative HEV was treated. When the positive hemisphere was treated there was an improvement of −2.3 (SD 2.6) on the 9-point OCS compared with an improvement of −1.6 (SD 2.2) points when the negative hemisphere was treated, which by a one-sided Wilcoxon test had a p = 0.0087. During the sham treatments there were some placebo improvements on both the correct and the incorrect hemispheres, but the difference between them was not significant by a one-sided Wilcoxon test, p = 0.446. The hemispheric differences between the active and the sham conditions were significant, p = 0.0437, (**Figure 6**).

### Opioid Use

We counted the number of urine drug screens that were positive for morphine or opioids and found five were positive for either at baseline, and of these four were in the Active–Sham group and none of the five were positive at either week 2 or 3. Two patients who were negative at baseline were positive at week 2 or at week 3. One was after an active treatment and one after a sham.

Using the timeline follow-back scores we found:

- 14/22 patients had zero opioid use at baseline over the preceding 7 days
- two of the 14 patients had on-study usage—both at week 2 after receiving sham treatment at week 1. They returned to zero at the week 3 assessment after receiving active treatment at week 2
- 12 of the 14 had no on-study usage
- Among the eight users at baseline (numbers in parentheses are quantitative data calculated from the followback timeline, not patient numbers)
- one patient was a “heavy” user at baseline (126). He received the active treatment at week 1 and saw his usage drop to (1.5) after the active treatment and then (zero) the following week
- four patients were “moderate” users at baseline (7, 14, 14, 8). All received active treatment at week 1, and all saw a decrease in usage at the week 2 assessment. Two of the four saw another decrease at the week 3 assessment. Another patient did not have a week 3 assessment while the fourth patient increased (8 at week 1, 6 at week 2, 10 at week 3, after sham at week 2).
- three patients were “low” users at baseline (1 or 2) and remained low users at each of the subsequent assessments

### Adverse Side-Effects

We purposely did not use a formal side-effect questionnaire to avoid negatively influencing the patients. All but one patient had a very positive relationship with the treatment team. The one exception was the patient mentioned above who complained of abdominal bloating the night of the active treatment. We know of one other report of a patient treated with t-PBM experiencing abdominal bloating possibly in response to t-PBM (37), who recovered rapidly.

## Discussion

The opioid crisis is well known and widely appreciated (1–4), but while current treatments can be effective when skillfully applied by both the patient and caregivers, they still leave a profound need for further improvements. We have developed a novel treatment that attempts to address the underlying depression and anxiety symptoms likely arising from unappreciated (or appreciated) past traumas and which can be both promoters of the addiction and consequences of it. The treatment tested in this study is based on DBP which suggests that in a large majority of patients, one hemisphere (left or right) is a trait associated with a mental state that manifests a relationship to past traumas (5, 20, 22, 25) causing depression, anxiety, and an immature cognition that leads to adverse behavior, including substance abuse. DBP also suggests that the opposite positive hemisphere is less affected by past trauma and is less prone to negative affects and behaviors (20). Based on Teicher’s (49, 50) work suggesting that the neuropsychiatric consequences of maltreatment relate to its type and timing (sensitive periods) in development, we suspect but have not demonstrated that the development of the negative, more traumatized hemisphere may also relate to these factors: type and timing. We have suggested that unilateral treatments to the positive hemisphere (5, 28, 29) might stimulate it and further promote its dominance thereby inducing a state of greater wellbeing. DBP therapy focuses on using the healthier hemisphere to assist the troubled side, like a parent, with love, insight, and limits. Our innovation is to combine DBP with unilateral tPBM to stimulate the healthy hemisphere with NIR light. Our results, documented in this paper, indicate that this new treatment, compared to sham, had an effect size of −.73, and the benefits were usually obvious to the patients and to our staff. A recent publication in the American Journal of Psychiatry described using CBD to reduce opioid cravings. They found a 3 to 5% decrease in craving in CBD group which was superior to placebo (51). We now have a follow-up blind, placebo-controlled trial offering patients twice a week treatments for 4 weeks underway, which was funded by a NIDA/SBIR grant (#1R43DA050358-01).

The mechanisms of tPBM have been well reviewed in many papers (5, 6, 8, 12) and include the absorption of photons by cytochrome-c to stimulate ATP formation (11) in the mitochondria, increased cerebral blood flow (5), decreased inflammatory factors (52), and increased brain neurotropic factors (53), but none of these mechanisms explains our observations of the often profound decreases in cravings, anxiety, and depression within minutes after a treatment has begun. Caldieraro and Cassano have speculated that tPBM might act through induced electromagnetic fields (54), and Schiffer has speculated that these changes might involve quantum effects due to alterations in biophoton emissions from tubulin molecules encoding brain information related to experiences (55), but these speculations have not yet been tested. What is clear, however, is that several studies have reported psychological benefits from bilateral tPBM (9, 37, 42, 44), including one case report of a 31-month treatment (56).

Because of his success in accurately predicting patients’ responses to left-sided rTMS using his DBP hypotheses, Schiffer was motivated to treat the right hemisphere with 10 Hz in patients in whom the visual test suggested that their right hemisphere was more positive, but he was unable to find an interested collaborator. His first study of tPBM was a bilateral pilot study of 10 patients with a history of trauma, anxiety and depression, seven of whom have a past history of substance abuse. All had been in remission for at least 4 years, and none had active cravings (5). Immediately after treatment and in follow-up these patients did not notice the improvement that was observed on the Hamilton Scales. Schiffer then used unilateral t-PBM in his private practice as an off-label treatment and found that the unilateral treatment to the positive hemisphere seemed to be far superior to the bilateral application as shown in **Table 8**. This paper reports the first double-blind, placebo-controlled study of unilateral t-PBM to a hemisphere with a more positive HEV. That unilateral tPBM to the positive hemisphere is superior to that to the negative hemisphere, and the fact that unilateral tPBM was effective in reducing cravings, depression, and anxiety offer support for Schiffer’s hypotheses as expressed in DBP and for his clinical observations from his practice and his research indicating that unilateral tPBM is superior to bilateral tPBM. Further study needs to directly compare unilateral and bilateral tPBM.

**Table 8:** Comparison of Absolute Change in Mean OCS Scores Between Active and Sham Treatment by Correct *vs.* Incorrect Hemisphere.

Statistic	Active Correct	Active Incorrect	Active Difference (Correct - Incorrect)	Sham Correct	Sham Incorrect	Sham Difference (Correct - Incorrect)	Difference of Differences (Active - Sham)
n	20	20	20	20	20	20	20
Mean (SD)	−2.3 (2.6)	−1.6 (2.2)	−0.7 (1.3)	−2.1 (2.2)	−1.9 (1.9)	−0.2 (1.4)	−0.5 (1.2)
Median	−2.7	−1.7	−0.3	−1.3	−2.0	0.0	−0.2
Min, Max	−8.0, 2.0	−6.0, 2.7	−4.7, 1.0	−7.3, 0.7	−6.7, 0.7	−5.0, 2.3	−2.7, 1.7
95% CI	(−3.5, −1.0)	(−2.6, −0.6)	(−1.2, −0.1)	(−3.1, −1.1)	(−2.8, −1.0)	(−0.8, 0.5)	(−1.0, 0.1)
p-value (paired t-test)			0.0155			0.2836	0.0431
p-value (signed-rank test)			0.0087			0.4462	0.0437

All Randomized Patients with Complete Data

[1] Wilcoxon signed-rank tests were performed to assess change from baseline in the OCS score on the correct and incorrect hemispheres after active treatment and sham treatment on the same day, separately. The correct and incorrect hemispheres were based on a computer test for emotional valance.

[2] The baseline score used to calculate absolute change corresponded to the pre-treatment score the week they received the assigned treatment. For patients who received active treatment at week 1, the baseline score from week 1 was used. For patients who received active treatment at week 2, the baseline score from week 2 was used. The baseline for sham treatment was derived in a similar way.

[3] A Wilcoxon signed-rank test was performed to compare the difference in absolute change from baseline in the OCS score on active treatment versus sham treatment (Difference of Differences column).

[4] One-sided lower p-values were calculated for all tests.

### Limitations

The main limitation of the study is its small number of patients and its short duration. t-PBM is a relatively new field, and many initial studies have had small numbers (9, 37, 56–58), and larger and longer studies are needed. We have completed 31 of 40 patients in a NIDA sponsored double-blind RTC study (Grant #1R43DA050358-01), which has been temporarily interrupted by the pandemic, but to date shows a highly significant treatment * time effect in regard to opioid craving reduction.

## Data Availability Statement

All datasets presented in this study are included in the article/**Supplementary Material**.

## Ethics Statement

The studies involving human participants were reviewed and approved by New England IRB, Needham, MA WIRB-Copernicus IRB Group. The patients/participants provided their written informed consent to participate in this study.

## Author Contributions

FS made substantial contributions to the conception or design of the work as well as to the acquisition, analysis or interpretation of data for the work; drafting the work or revising it critically for important intellectual content. WR performed the statistical analyses for the work. EF and HM made substantial contributions to the acquisition of data for the work. MH made substantial contributions to the conception or design of the work; analysis or interpretation of data for the work and revising it critically for important intellectual content. All authors have provided approval for publication of the content, and agreed to be accountable for all aspects of the work in ensuring that questions related to the accuracy or integrity of any part of the work are appropriately investigated and resolved.

## Conflict of Interest

WR was a freelance statistician who was referred to MindLight by Horizon Data Science Applications. FS is Founder and President of MindLight, LLC, which has been awarded a NIDA/SBIR grant (1 R43 DA050358-01) for further research and commercialization of the methods and device described in the paper. FS has been issued 2 US patents which cover the method of unilateral tPBM to a positive hemisphere as described in this study: U.S. Patent No. 8303636, Issued 11/06/2012: Methods for treating psychiatric disorders using light energy; and U.S. Patent No. 8574279, Issued 11/05/2013: Methods for treating psychiatric disorders using light energy. FS has filed on December 5, 2019, a US patent application, #16/703,937, Method and Apparatus for Determining Hemispheric Emotional Valence.

The remaining authors declare that the research was conducted in the absence of any commercial or financial relationships that could be construed as a potential conflict of interest.
