# Supplementary material for: A Novel Treatment of Opioid Cravings With an Effect Size of .73 for Unilateral Transcranial Photobiomodulation Over Sham
Source: Front Psychiatry. 2020 Aug 19;11:827. doi: 10.3389/fpsyt.2020.00827 (PMC7466767; doi:10.3389/fpsyt.2020.00827)
Supplement: Supplementary file 1 [file DataSheet_1.docx]

Table 1. Baseline Demographic and Clinical Characteristics All Randomized Patients with Complete Data **(Table 1. In the Paper)**

| **Characteristic** | **Active-Sham (n=9)** | **Sham-Active (n=13)** | **All Patients (n=22)** |
| --- | --- | --- | --- |
| Age (years) |  |  |  |
| n | 9 | 13 | 22 |
| Mean (SD) | 52.6 (9.8) | 46.5 (9.2) | 49.0 (9.7) |
| Median | 55.0 | 45.0 | 51.5 |
| Min, Max | 31.0, 63.0 | 34.0, 62.0 | 31.0, 63.0 |
|  |  |  |  |
| Gender |  |  |  |
| Male | 8 (88.9%) | 12 (92.3%) | 20 (90.9%) |
| Female | 1 (11.1%) | 1 (7.7%) | 2 (9.1%) |
|  |  |  |  |
| Race/Ethnicity |  |  |  |
| Black | 6 (66.7%) | 4 (30.8%) | 10 (45.5%) |
| White | 3 (33.3%) | 8 (61.5%) | 11 (50.0%) |
| Black Hispanic | 0 (0.0%) | 1 (7.7%) | 1 (4.5%) |
|  |  |  |  |
| Highest Education Grade |  |  |  |
| 4 | 1 (11.1%) | 0 (0.0%) | 1 (4.5%) |
| 10 | 1 (11.1%) | 2 (15.4%) | 3 (13.6%) |
| 12 | 4 (44.4%) | 3 (23.1%) | 7 (31.8%) |
| 13 | 0 (0.0%) | 3 (23.1%) | 3 (13.6%) |
| 14 | 3 (33.3%) | 4 (30.8%) | 7 (31.8%) |
| 16 | 0 (0.0%) | 1 (7.7%) | 1 (4.5%) |
|  |  |  |  |
| ACE Score |  |  |  |
| n | 9 | 13 | 22 |
| Mean (SD) | 4.6 (3.3) | 3.1 (2.8) | 3.7 (3.0) |
| Median | 3.0 | 2.0 | 2.5 |
| Min, Max | 1.0, 9.0 | 0.0, 8.0 | 0.0, 9.0 |
|  |  |  |  |
| Handedness |  |  |  |
| Left | 2 (22.2%) | 1 (7.7%) | 3 (13.6%) |
| Right | 7 (77.8%) | 12 (92.3%) | 19 (86.4%) |
|  |  |  |  |
| Baseline HDRS |  |  |  |
| n | 9 | 13 | 22 |
| Mean (SD) | 9.9 (11.4) | 14.1 (13.4) | 12.4 (12.5) |
| Median | 6.0 | 8.0 | 7.0 |
| Min, Max | 1.0, 35.0 | 2.0, 41.0 | 1.0, 41.0 |
|  |  |  |  |
| Baseline HARS |  |  |  |
| n | 9 | 13 | 22 |
| Mean (SD) | 12.8 (16.8) | 11.8 (12.1) | 12.2 (13.8) |
| Median | 7.0 | 6.0 | 6.5 |
| Min, Max | 0.0, 51.0 | 1.0, 32.0 | 0.0, 51.0 |
|  |  |  |  |
| Baseline PANAS (Positive) |  |  |  |
| n | 9 | 13 | 22 |
| Mean (SD) | 15.4 (5.9) | 16.2 (4.6) | 15.9 (5.1) |
| Median | 17.0 | 16.0 | 16.5 |
| Min, Max | 5.0, 23.0 | 7.0, 25.0 | 5.0, 25.0 |
|  |  |  |  |
| Baseline PANAS (Negative) |  |  |  |
| n | 9 | 13 | 22 |
| Mean (SD) | 11.1 (6.2) | 8.5 (3.6) | 9.5 (4.9) |
| Median | 7.0 | 7.0 | 7.0 |
| Min, Max | 5.0, 21.0 | 5.0, 16.0 | 5.0, 21.0 |
|  |  |  |  |

Table 2. Comparison of Mean OCS Scores Between Active and Sham Treatment (Same Day Analysis)

All Randomized Patients with Complete Data **(Table 2 in the Paper)**

| **Statistic** | **Baseline (Week 1)** | **Active Post-Treatment** | **Difference (Active - Baseline)** | **Sham Post-Treatment** | **Difference (Sham - Baseline)** | **Difference of Differences (Active - Sham)** |
| --- | --- | --- | --- | --- | --- | --- |
| n | 20 | 20 | 20 | 20 | 20 | 20 |
| Mean (SD) | 6.8 (1.5) | 3.9 (2.9) | -2.8 (2.7) | 3.8 (2.6) | -3.0 (2.5) | 0.1 (2.9) |
| Median | 7.0 | 3.7 | -2.8 | 3.7 | -2.5 | -0.2 |
| Min, Max | 3.7, 9.0 | 0.0, 9.0 | -8.3, 2.3 | 0.0, 9.0 | -8.3, 0.7 | -7.3, 5.7 |
| 95% CI | (6.1, 7.5) | (2.6, 5.3) | (-4.1, -1.6) | (2.6, 5.0) | (-4.2, -1.8) | (-1.2, 1.5) |
| p-value |  |  | <.0001 |  | <.0001 | 0.5856 |

[1] Paired t-tests were performed to assess change from baseline in the mean OCS score after active treatment and sham treatment, separately.

[2] A paired t-test was performed to compare the change from baseline in the mean OCS score on active treatment versus sham treatment (Difference of Differences column). A positive value for the difference of difference favors sham treatment, while a negative value favors active treatment.

[3] One-sided lower p-values were calculated for all tests.

Table 2. Comparison of Mean OCS Scores (Percent Change) Between Active and Sham Treatment (Same Day Analysis)

All Randomized Patients with Complete Data

| **Statistic** | **Baseline (Week 1)** | **Active Post-Treatment** | **Pct Difference (Active - Baseline)** | **Sham Post-Treatment** | **Pct Difference (Sham - Baseline)** | **Difference of Pct Differences (Active - Sham)** |
| --- | --- | --- | --- | --- | --- | --- |
| n | 20 | 20 | 20 | 20 | 20 | 20 |
| Mean (SD) | 6.8 (1.5) | 3.9 (2.9) | -44.1 (39.6) | 3.8 (2.6) | -45.5 (35.6) | 1.4 (39.0) |
| Median | 7.0 | 3.7 | -47.6 | 3.7 | -43.3 | -2.4 |
| Min, Max | 3.7, 9.0 | 0.0, 9.0 | -100, 38.9 | 0.0, 9.0 | -100, 8.0 | -88.0, 77.8 |
| 95% CI | (6.1, 7.5) | (2.6, 5.3) | (-62.7, -25.6) | (2.6, 5.0) | (-62.2, -28.9) | (-16.8, 19.6) |
| p-value |  |  | <.0001 |  | <.0001 | 0.5625 |

[1] Paired t-tests were performed to assess percent change from baseline in the mean OCS score after active treatment and sham treatment, separately.

[2] A paired t-test was performed to compare the percent change from baseline in the mean OCS score on active treatment versus sham treatment (Difference of Pct Differences column). A positive value for the difference of percent difference favors sham treatment, while a negative value favors active treatment.

[3] One-sided lower p-values were calculated for all tests.

Table 3. Comparison of Mean OCS Scores Between Active and Sham Treatment Using Baseline Score at the Corresponding Week of Treatment (Same Day Analysis)

All Randomized Patients with Complete Data

| **Statistic** | **Baseline Active** | **Active Post-Treatment** | **Difference (Active - Baseline)** | **Baseline Sham** | **Sham Post-Treatment** | **Difference (Sham - Baseline)** | **Difference of Differences (Active - Sham)** |
| --- | --- | --- | --- | --- | --- | --- | --- |
| n | 20 | 20 | 20 | 20 | 20 | 20 | 20 |
| Mean (SD) | 6.2 (2.3) | 3.9 (2.9) | -2.3 (2.6) | 5.9 (2.1) | 3.8 (2.6) | -2.1 (2.2) | -0.2 (3.0) |
| Median | 7.0 | 3.7 | -2.7 | 6.0 | 3.7 | -1.3 | -0.5 |
| Min, Max | 2.0, 9.0 | 0.0, 9.0 | -8.0, 2.0 | 0.0, 8.3 | 0.0, 9.0 | -7.3, 0.7 | -5.0, 6.0 |
| 95% CI | (5.1, 7.3) | (2.6, 5.3) | (-3.5, -1.0) | (4.9, 6.9) | (2.6, 5.0) | (-3.1, -1.1) | (-1.6, 1.2) |
| p-value |  |  | 0.0005 |  |  | 0.0002 | 0.4018 |

[1] Paired t-tests were performed to assess change from baseline in the mean OCS score after active treatment and sham treatment on the same day, separately. The baseline score used corresponded to the week they received the assigned treatment. For patients who received active treatment at week 1, the baseline score from week 1 was used. For patients who received active treatment at week 2, the baseline score from week 2 was used. The baseline for sham treatment was derived in a similar way.

[2] A paired t-test was performed to compare the change from baseline in the mean OCS score on active treatment versus sham treatment (Difference of Differences column). A positive value for the difference of difference favors sham treatment, while a negative value favors active treatment

[3] One-sided lower p-values were calculated for all tests.

Table 3.1. Comparison of Mean OCS Scores (Percent Change) Between Active and Sham Treatment Using Baseline Score at the Corresponding Week of Treatment (Same Day Analysis)

All Randomized Patients with Complete Data

| **Statistic** | **Baseline Active** | **Active Post-Treatment** | **Pct Difference (Active - Baseline)** | **Baseline Sham** | **Sham Post-Treatment** | **Pct Difference (Sham - Baseline)** | **Difference of Pct Differences (Active - Sham)** |
| --- | --- | --- | --- | --- | --- | --- | --- |
| n | 20 | 20 | 20 | 20 | 20 | 20 | 20 |
| Mean (SD) | 6.2 (2.3) | 3.9 (2.9) | -34.6 (46.3) | 5.9 (2.1) | 3.8 (2.6) | -36.4 (33.8) | 1.8 (48.2) |
| Median | 7.0 | 3.7 | -37.8 | 6.0 | 3.7 | -28.2 | -2.8 |
| Min, Max | 2.0, 9.0 | 0.0, 9.0 | -100, 66.7 | 0.0, 8.3 | 0.0, 9.0 | -100, 10.0 | -100, 81.0 |
| 95% CI | (5.1, 7.3) | (2.6, 5.3) | (-56.2, -12.9) | (4.9, 6.9) | (2.6, 5.0) | (-52.2, -20.6) | (-20.7, 24.4) |
| p-value |  |  | 0.0017 |  |  | <.0001 | 0.5660 |

[1] Paired t-tests were performed to assess percent change from baseline in the mean OCS score after active treatment and sham treatment on the same day, separately. The baseline score used corresponded to the week they received the assigned treatment. For patients who received active treatment at week 1, the baseline score from week 1 was used. For patients who received active treatment at week 2, the baseline score from week 2 was used. The baseline for sham treatment was derived in a similar way.

[2] A paired t-test was performed to compare the pecent change from baseline in the mean OCS score on active treatment versus sham treatment (Difference of Pct Differences column). A positive value for the difference of percent difference favors sham treatment, while a negative value favors active treatment

[3] One-sided lower p-values were calculated for all tests.

Table 4. Comparison of Mean OCS Scores Between Active and Sham Treatment at Baseline (Week 1) and Follow-up (Week 2)

All Randomized Patients with Complete Data

| **Statistic** | **Active** | **Sham** | **Difference** |
| --- | --- | --- | --- |
| Week 1 (Baseline) |  |  |  |
| n | 7 | 13 |  |
| Mean (SD) | 7.2 (1.8) | 6.5 (1.3) | 0.7 (1.5) |
| 95% CI | (5.6, 8.9) | (5.7, 7.3) | (-0.8, 2.2) |
| p-value |  |  | 0.8378 |
|  |  |  |  |
| Week 2 (Follow-up) |  |  |  |
| n | 7 | 13 |  |
| Mean (SD) | 4.7 (2.8) | 5.6 (2.3) | -0.9 (2.5) |
| 95% CI | (2.1, 7.3) | (4.2, 7.1) | (-3.4, 1.6) |
| p-value |  |  | 0.2214 |
|  |  |  |  |
| Week 2 - Week 1 (Absolute) |  |  |  |
| n | 7 | 13 |  |
| Mean (SD) | -2.5 (2.2) | -0.9 (1.9) | -1.6 (2.0) |
| 95% CI | (-4.6, -0.5) | (-2.0, 0.2) | (-3.6, 0.3) |
| p-value |  |  | 0.0476 |
|  |  |  |  |
| Week 2 - Week 1 (Percent) |  |  |  |
| n | 7 | 13 |  |
| Mean (SD) | -39.0 (35.8) | -14.4 (32.1) | -24.5 (33.4) |
| 95% CI | (-72.1, -5.8) | (-33.8, 5.0) | (-57.4, 8.4) |
| p-value |  |  | 0.0673 |
|  |  |  |  |

[1] Both the baseline and follow-up OCS scores used in this analysis were assessed prior to the start of treatment for that week -- i.e. the OCS score at Week 2 represents the level of cravings 1 week after receiving the first treatment in the treatment sequence.

[2] Columns for Active and Sham treatment are based on the first treatment in the treatment sequence the patient was randomized to.

[3] Independent samples t-tests were performed to assess differences in OCS at baseline (prior to any treatment), follow-up (after first treatment in the randomized sequence), and change from baseline by treatment received at Week 1.

[4] Higher values at Week 1 and Week 2 indicate increased craving. Negative values for the change score indicate a decrease in cravings. Positive values for the difference between groups indicate more cravings associated with Active treatment, while negative values indicate less cravings associated with Active treatment.

[5] One-sided lower p-values were calculated for all tests.

Table 5. Comparison of Mean OCS Scores Between Active and Sham Treatment at Baseline (Week 1) and Follow-up (Week 3)

All Randomized Patients with Complete Data

| **Statistic** | **Active (after Sham)** | **Sham (after Active)** | **Difference** |
| --- | --- | --- | --- |
| Week 1 (Baseline) |  |  |  |
| n | 12 | 5 |  |
| Mean (SD) | 6.6 (1.4) | 7.6 (0.8) | -1.0 (1.3) |
| 95% CI | (5.7, 7.5) | (6.6, 8.6) | (-2.4, 0.4) |
| p-value |  |  | 0.0744 |
|  |  |  |  |
| Week 3 (Follow-up) |  |  |  |
| n | 12 | 5 |  |
| Mean (SD) | 2.4 (2.3) | 5.7 (3.5) | -3.3 (2.7) |
| 95% CI | (1.0, 3.9) | (1.4, 10.1) | (-6.4, -0.3) |
| p-value |  |  | 0.0176 |
|  |  |  |  |
| Week 3 - Week 1 (Absolute) |  |  |  |
| n | 12 | 5 |  |
| Mean (SD) | -4.2 (2.3) | -1.9 (3.1) | -2.3 (2.5) |
| 95% CI | (-5.6, -2.7) | (-5.8, 2.0) | (-5.2, 0.6) |
| p-value |  |  | 0.0552 |
|  |  |  |  |
| Week 3 - Week 1 (Percent) |  |  |  |
| n | 12 | 5 |  |
| Mean (SD) | -64.3 (30.8) | -25.8 (43.9) | -38.5 (34.8) |
| 95% CI | (-83.9, -44.8) | (-80.3, 28.7) | (-77.9, 0.9) |
| p-value |  |  | 0.0275 |
|  |  |  |  |

[1] Both the baseline and follow-up OCS scores used in this analysis were assessed prior to the start of treatment for that week -- i.e. the OCS score at Week 3 represents the level of cravings 1 week after receiving the second treatment in the treatment sequence.

[2] Columns for Active and Sham treatment are based on the second treatment in the treatment sequence the patient was randomized to.

[3] Independent samples t-tests were performed to assess differences in OCS at baseline (prior to any treatment), follow-up (after second treatment in the randomized sequence), and change from baseline by treatment received at Week 2.

[4] Higher values at Week 1 and Week 3 indicate increased craving. Negative values for the change score indicate a decrease in cravings. Positive values for the difference between groups indicate more cravings associated with Active treatment, while negative values indicate less cravings associated with Active treatment.

[5] One-sided lower p-values were calculated for all tests.

Table 6. Comparison of Mean OCS Scores Between Active and Sham Treatment at Baseline (Week 2) and Follow-up (Week 3)

All Randomized Patients with Complete Data

| **Statistic** | **Active (after Sham)** | **Sham (after Active)** | **Difference** |
| --- | --- | --- | --- |
| Week 2 (Baseline) |  |  |  |
| n | 12 | 5 |  |
| Mean (SD) | 5.9 (2.3) | 6.2 (1.4) | -0.3 (2.1) |
| 95% CI | (4.4, 7.3) | (4.5, 7.9) | (-2.7, 2.0) |
| p-value |  |  | 0.3828 |
|  |  |  |  |
| Week 3 (Follow-up) |  |  |  |
| n | 12 | 5 |  |
| Mean (SD) | 2.4 (2.3) | 5.7 (3.5) | -3.3 (2.7) |
| 95% CI | (1.0, 3.9) | (1.4, 10.1) | (-6.4, -0.3) |
| p-value |  |  | 0.0176 |
|  |  |  |  |
| Week 3 - Week 2 (Absolute) |  |  |  |
| n | 12 | 5 |  |
| Mean (SD) | -3.4 (2.8) | -0.5 (2.7) | -3.0 (2.8) |
| 95% CI | (-5.2, -1.6) | (-3.8, 2.9) | (-6.1, 0.2) |
| p-value |  |  | 0.0317 |
|  |  |  |  |
| Week 3 - Week 2 (Percent) |  |  |  |
| n | 12 | 5 |  |
| Mean (SD) | -54.7 (33.1) | -11.2 (49.3) | -43.5 (38.1) |
| 95% CI | (-75.7, -33.6) | (-72.4, 50.0) | (-86.7, -0.3) |
| p-value |  |  | 0.0244 |
|  |  |  |  |

[1] Both the baseline and follow-up OCS scores used in this analysis were assessed prior to the start of treatment for that week -- i.e. the OCS score at Week 3 represents the level of cravings 1 week after receiving the second treatment in the treatment sequence.

[2] Columns for Active and Sham treatment are based on the second treatment in the treatment sequence the patient was randomized to.

[3] Independent samples t-tests were performed to assess differences in OCS at baseline (1 week after first treatment but before the second treatment), follow-up (after second treatment in the randomized sequence), and change from baseline by treatment received at Week 2.

[4] Higher values at Week 2 and Week 3 indicate increased craving. Negative values for the change score indicate a decrease in cravings. Positive values for the difference between groups indicate more cravings associated with Active treatment, while negative values indicate less cravings associated with Active treatment.

[5] One-sided lower p-values were calculated for all tests.

Table 7. Comparison of Mean OCS Scores Between Active and Sham Treatment One Week After Treatment

All Randomized Patients with Complete Data

| **Statistic** | **Baseline (Week 1)** | **Active** | **Difference (Active - Baseline)** | **Sham** | **Difference (Sham - Baseline)** | **Difference of Differences (Active - Sham)** |
| --- | --- | --- | --- | --- | --- | --- |
| n | 17 | 17 | 17 | 17 | 17 | 17 |
| Mean (SD) | 6.9 (1.3) | 3.5 (2.7) | -3.3 (2.3) | 5.8 (2.6) | -1.1 (2.3) | -2.3 (3.3) |
| Median | 7.0 | 3.3 | -3.7 | 6.7 | -0.3 | -2.3 |
| Min, Max | 4.3, 9.0 | 0.0, 8.3 | -8.3, 0.0 | 2.0, 9.0 | -5.3, 2.0 | -8.0, 3.7 |
| 95% CI | (6.2, 7.6) | (2.1, 4.9) | (-4.5, -2.2) | (4.5, 7.2) | (-2.2, 0.1) | (-4.0, -0.6) |
| p-value |  |  | <.0001 |  | 0.0353 | 0.0053 |

[1] Paired t-tests were performed to assess change from baseline in the mean OCS score one week after active treatment and sham treatment, separately.

[2] A paired t-test was performed to compare the change from baseline in the mean OCS score on active treatment versus sham treatment (Difference of Differences column). A positive value for the difference of difference favors sham treatment, while a negative value favors active treatment.

[3] One-sided lower p-values were calculated for all tests.

Table 7.1. Comparison of Mean OCS Scores (Percent Change) Between Active and Sham Treatment One Week After Treatment

All Randomized Patients with Complete Data **(Table 3. In the Paper)**

| **Statistic** | **Baseline (Week 1)** | **Active** | **Pct Difference (Active - Baseline)** | **Sham** | **Pct Difference (Sham - Baseline)** | **Difference of Pct Differences (Active - Sham)** |
| --- | --- | --- | --- | --- | --- | --- |
| n | 17 | 17 | 17 | 17 | 17 | 17 |
| Mean (SD) | 6.9 (1.3) | 3.5 (2.7) | -51.0 (33.7) | 5.8 (2.6) | -15.8 (35.0) | -35.2 (48.2) |
| Median | 7.0 | 3.3 | -60.0 | 6.7 | -4.2 | -31.8 |
| Min, Max | 4.3, 9.0 | 0.0, 8.3 | -100, 0.0 | 2.0, 9.0 | -72.7, 33.3 | -115, 52.4 |
| 95% CI | (6.2, 7.6) | (2.1, 4.9) | (-68.3, -33.7) | (4.5, 7.2) | (-33.7, 2.2) | (-60.0, -10.5) |
| p-value |  |  | <.0001 |  | 0.0408 | 0.0041 |

[1] Paired t-tests were performed to assess percent change from baseline in the mean OCS score one week after active treatment and sham treatment, separately.

[2] A paired t-test was performed to compare the percent change from baseline in the mean OCS score on active treatment versus sham treatment (Difference of Percent Differences column). A positive value for the difference of percent difference favors sham treatment, while a negative value favors active treatment.

[3] One-sided lower p-values were calculated for all tests.

Table 8. Comparison of Mean OCS Scores Between Active and Sham Treatment Using the OCS Score from the Prior Week as the Baseline Score

All Randomized Patients with Complete Data

| **Statistic** | **Baseline Active** | **Active One Week Later** | **Difference (Active - Baseline)** | **Baseline Sham** | **Sham One Week Later** | **Difference (Sham - Baseline)** | **Difference of Differences (Active - Sham)** |
| --- | --- | --- | --- | --- | --- | --- | --- |
| n | 17 | 17 | 17 | 17 | 17 | 17 | 17 |
| Mean (SD) | 6.4 (2.1) | 3.5 (2.7) | -2.8 (2.5) | 6.5 (1.4) | 5.8 (2.6) | -0.6 (2.0) | -2.2 (3.8) |
| Median | 7.0 | 3.3 | -2.3 | 6.3 | 6.7 | -0.3 | -1.3 |
| Min, Max | 2.0, 9.0 | 0.0, 8.3 | -8.0, 0.3 | 4.3, 8.3 | 2.0, 9.0 | -3.7, 3.0 | -8.7, 2.6 |
| 95% CI | (5.3, 7.5) | (2.1, 4.9) | (-4.1, -1.5) | (5.8, 7.2) | (4.5, 7.2) | (-1.7, 0.4) | (-4.1, -0.3) |
| p-value |  |  | 0.0002 |  |  | 0.1050 | 0.0143 |

[1] Paired t-tests were performed to assess change from baseline in the mean OCS score one week after active treatment and sham treatment, separately. The baseline score used corresponded to the pre-treatment score from the prior week. For patients who received active treatment at week 1, the pre-treatment score at week 1 was the baseline and the pre-treatment score at week 2 was the follow-up. For patients who received active treatment at week 2, the pre-treatment score at week 2 was the baseline and the pre-treatment score at week 3 was the follow-up. The baseline and follow-up scores for sham treatment were derived in a similar way.

[2] A paired t-test was performed to compare the change from baseline in the mean OCS score on active treatment versus sham treatment (Difference of Differences column). A positive value for the difference of difference favors sham treatment, while a negative value favors active treatment

[3] One-sided lower p-values were calculated for all tests.

Table 8.1. Comparison of Mean OCS Scores (Percent) Between Active and Sham Treatment Using the OCS Score from the Prior Week as the Baseline Score

All Randomized Patients with Complete Data **(Table 4 in the Paper)**

| **Statistic** | **Baseline Active** | **Active One Week Later** | **Pct Difference (Active - Baseline)** | **Baseline Sham** | **Sham One Week Later** | **Pct Difference (Sham - Baseline)** | **Difference of Pct Differences (Active - Sham)** |
| --- | --- | --- | --- | --- | --- | --- | --- |
| n | 17 | 17 | 17 | 17 | 17 | 17 | 17 |
| Mean (SD) | 6.4 (2.1) | 3.5 (2.7) | -44.2 (32.6) | 6.5 (1.4) | 5.8 (2.6) | -11.5 (36.1) | -32.7 (53.7) |
| Median | 7.0 | 3.3 | -36.4 | 6.3 | 6.7 | -4.0 | -18.0 |
| Min, Max | 2.0, 9.0 | 0.0, 8.3 | -100, 4.3 | 4.3, 8.3 | 2.0, 9.0 | -64.7, 52.9 | -115, 45.7 |
| 95% CI | (5.3, 7.5) | (2.1, 4.9) | (-61.0, -27.4) | (5.8, 7.2) | (4.5, 7.2) | (-30.0, 7.1) | (-60.3, -5.1) |
| p-value |  |  | <.0001 |  |  | 0.1046 | 0.0115 |

[1] Paired t-tests were performed to assess percent change from baseline in the mean OCS score one week after active treatment and sham treatment, separately. The baseline score used corresponded to the pre-treatment score from the prior week. For patients who received active treatment at week 1, the pre-treatment score at week 1 was the baseline and the pre-treatment score at week 2 was the follow-up. For patients who received active treatment at week 2, the pre-treatment score at week 2 was the baseline and the pre-treatment score at week 3 was the follow-up. The baseline and follow-up scores for sham treatment were derived in a similar way.

[2] A paired t-test was performed to compare the percent change from baseline in the mean OCS score on active treatment versus sham treatment (Difference of Percent Differences column). A positive value for the difference of percent difference favors sham treatment, while a negative value favors active treatment

[3] One-sided lower p-values were calculated for all tests.

Table 9.1a. Summary of Repeated Measures Model for Absolute Change in Mean OCS

Table All Randomized Patients Non-Missing Data at Particular Visit

Summary of Parameter (Beta) Estimates from Repeated Measures Model

| **Parameter** | **Beta Estimate (Std Err)** | **95% CI** | **P-value** |
| --- | --- | --- | --- |
| Intercept | -2.35 (0.80) | -4.03, -0.67 | 0.0088 |
| Active-Sham Sequence (vs Sham-Active Sequence) | 0.20 (0.88) | -1.66, 2.06 | 0.8216 |
| Active Treatment (vs Sham Treatment) | -1.84 (0.75) | -3.42, -0.27 | 0.0245 |
| Week 2 (vs Week 3) | 1.47 (0.75) | -0.11, 3.05 | 0.0666 |

[1] Repeated measures model for absolute change in mean OCS assessed prior to treatment each week that includes treatment, treatment sequence, and week as independent variables was constructed.

Table 9.1b. Summary of Repeated Measures Model for Absolute Change in Mean OCS

Table All Randomized Patients Non-Missing Data at Particular Visit

Summary of Adjusted Means by Treatment from the Repeated Measures Model

| **Treatment** | **Adjusted Mean (95% CI)** |
| --- | --- |
| Active | -3.36 (-4.46, -2.25) |
| Sham | -1.51 (-2.84, -0.19) |

[1] Repeated measures model for absolute change in mean OCS assessed prior to treatment each week that includes treatment, treatment sequence, and week as independent variables was constructed.

Table 9.2a. Summary of Repeated Measures Model for Percent Change in Mean OCS

Table All Randomized Patients Non-Missing Data at Particular Visit

Summary of Parameter (Beta) Estimates from Repeated Measures Model

| **Parameter** | **Beta Estimate (Std Err)** | **95% CI** | **P-value** |
| --- | --- | --- | --- |
| Intercept | -35.14 (11.74) | -59.80, -10.48 | 0.0078 |
| Active-Sham Sequence (vs Sham-Active Sequence) | 5.22 (13.12) | -22.34, 32.78 | 0.6953 |
| Active Treatment (vs Sham Treatment) | -29.76 (10.92) | -53.04, -6.48 | 0.0157 |
| Week 2 (vs Week 3) | 20.73 (10.92) | -2.56, 44.01 | 0.0772 |

[1] Repeated measures model for percent change in mean OCS assessed prior to treatment each week that includes treatment, treatment sequence, and week as independent variables was constructed.

Table 9.2b. Summary of Repeated Measures Model for Percent Change in Mean OCS

Table All Randomized Patients Non-Missing Data at Particular Visit

Summary of Adjusted Means by Treatment from the Repeated Measures Model

| **Treatment** | **Adjusted Mean (95% CI)** |
| --- | --- |
| Active | -51.93 (-69.21, -34.64) |
| Sham | -22.17 (-41.22, -3.12) |

[1] Repeated measures model for percent change in mean OCS assessed prior to treatment each week that includes treatment, treatment sequence, and week as independent variables was constructed.

Table 10.1. Comparison of Absolute Change in Mean OCS Scores Between Active and Sham Treatment by Correct vs. Incorrect Hemisphere

All Randomized Patients with Complete Data

| **Statistic** | **Active Correct** | **Active Incorrect** | **Active Difference (Correct - Incorrect)** | **Sham Correct** | **Sham Incorrect** | **Sham Difference (Correct - Incorrect)** | **Difference of Differences (Active - Sham)** |
| --- | --- | --- | --- | --- | --- | --- | --- |
| n | 20 | 20 | 20 | 20 | 20 | 20 | 20 |
| Mean (SD) | -2.3 (2.6) | -1.6 (2.2) | -0.7 (1.3) | -2.1 (2.2) | -1.9 (1.9) | -0.2 (1.4) | -0.5 (1.2) |
| Median | -2.7 | -1.7 | -0.3 | -1.3 | -2.0 | 0.0 | -0.2 |
| Min, Max | -8.0, 2.0 | -6.0, 2.7 | -4.7, 1.0 | -7.3, 0.7 | -6.7, 0.7 | -5.0, 2.3 | -2.7, 1.7 |
| 95% CI | (-3.5, -1.0) | (-2.6, -0.6) | (-1.2, -0.1) | (-3.1, -1.1) | (-2.8, -1.0) | (-0.8, 0.5) | (-1.0, 0.1) |
| p-value |  |  | 0.0155 |  |  | 0.2836 | 0.0431 |

[1] Paired t-tests were performed to assess change from baseline in the mean OCS score on the correct and incorrect hemisphere after active treatment and sham treatment on the same day, separately. The correct and incorrect hemisphere was based on a computer test for emotional valance.

[2] The baseline score used to calculate absolute change corresponded to the pre-treatment score the week they received the assigned treatment. For patients who received active treatment at week 1, the baseline score from week 1 was used. For patients who received active treatment at week 2, the baseline score from week 2 was used. The baseline for sham treatment was derived in a similar way.

[3] A paired t-test was performed to compare the difference in absolute change from baseline in the mean OCS score on active treatment versus sham treatment (Difference of Differences column).

[4] One-sided lower p-values were calculated for all tests.

Table 10.2. Comparison of Percent Change in Mean OCS Scores Between Active and Sham Treatment by Correct vs. Incorrect Hemisphere

All Randomized Patients with Complete Data

| **Statistic** | **Active Correct** | **Active Incorrect** | **Active Difference (Correct - Incorrect)** | **Sham Correct** | **Sham Incorrect** | **Sham Difference (Correct - Incorrect)** | **Difference of Differences (Active - Sham)** |
| --- | --- | --- | --- | --- | --- | --- | --- |
| n | 19 | 19 | 19 | 19 | 19 | 19 | 19 |
| Mean (SD) | -31.1 (44.9) | -22.8 (41.7) | -8.4 (21.9) | -38.3 (33.6) | -34.4 (31.5) | -3.9 (21.8) | -4.5 (25.6) |
| Median | -37.5 | -20.8 | -8.3 | -35.3 | -31.4 | 0.0 | -8.3 |
| Min, Max | -100, 66.7 | -100, 88.9 | -58.3, 50.0 | -100, 10.0 | -92.9, 8.0 | -60.0, 31.8 | -39.4, 50.0 |
| 95% CI | (-52.7, -9.5) | (-42.9, -2.6) | (-18.9, 2.2) | (-54.5, -22.1) | (-49.6, -19.3) | (-14.4, 6.7) | (-16.8, 7.8) |
| p-value |  |  | 0.0564 |  |  | 0.2257 | 0.2264 |

[1] Paired t-tests were performed to assess percent change from baseline in the mean OCS score on the correct and incorrect hemisphere after active treatment and sham treatment on the same day, separately. The correct and incorrect hemisphere was based on a computer test for emotional valance.

[2] The baseline score used to calculate percent change corresponded to the pre-treatment score the week they received the assigned treatment. For patients who received active treatment at week 1, the baseline score from week 1 was used. For patients who received active treatment at week 2, the baseline score from week 2 was used. The baseline for sham treatment was derived in a similar way.

[3] A paired t-test was performed to compare the difference in percent change from baseline in the mean OCS score on active treatment versus sham treatment (Difference of Differences column).

[4] One-sided lower p-values were calculated for all tests.

Table 11.1. Simple Linear Regression Models of Difference in Percent Change in OCS from Baseline Between Active and Sham Treatment [1,2]

All Randomized Patients with Complete Data

| **Independent Variable** | **Parameter Estimate (SE)** | **95% Confidence Interval** | **P-value** |
| --- | --- | --- | --- |
| HDRS | -0.64 (0.90) | [-2.56, 1.28] | 0.4851 |
|  |  |  |  |
| HARS | -0.02 (0.82) | [-1.76, 1.72] | 0.9823 |
|  |  |  |  |
| PANAS Positive | 5.21 (2.18) | [0.57, 9.85] | 0.0303 |
|  |  |  |  |
| PANAS Negative | 0.18 (2.63) | [-5.44, 5.79] | 0.9479 |
|  |  |  |  |
| Well-Being | -12.07 (5.60) | [-24.38, 0.25] | 0.0540 |
|  |  |  |  |
| Distress | -1.17 (4.65) | [-11.41, 9.06] | 0.8057 |
|  |  |  |  |
| Distress/Craving Sum (Absolute Difference in Sides) via LVFT | -9.34 (7.87) | [-26.13, 7.44] | 0.2539 |
|  |  |  |  |
| Distress/Craving Sum (Absolute Difference in Sides) via CTHEV | -2.85 (2.40) | [-7.97, 2.28] | 0.2551 |
|  |  |  |  |

[1] Separate models were constructed examining one independent variable at a time. Baseline OCS was based on the Week 1 score for calculating percent change.

[2] The outcome is scaled as the difference in percent change from baseline. Positive values for the parameter estimate indicates that higher values of the independent variable are associated with more favorable response to sham treatment, while negative values for the parameter estimate indicates that higher values of the independent variable are associated with more favorable response to active treatment.

Abbreviations: HDRS - Hamilton Depression Rating Scale; HARS - Hamilton Anxiety Rating Scale; PANAS - Positive and Negative Affect Schedule; LVFT - Lateral Visual Field Test; CTHEV - Computerized Hemispheric Valence

Table 11.2. Simple Linear Regression Models of Difference in Percent Change in OCS from Baseline Between Active and Sham Treatment [1,2]

All Randomized Patients with Complete Data

| **Independent Variable** | **Parameter Estimate (SE)** | **95% Confidence Interval** | **P-value** |
| --- | --- | --- | --- |
| HDRS | -0.67 (1.01) | [-2.81, 1.48] | 0.5176 |
|  |  |  |  |
| HARS | -0.14 (0.91) | [-2.07, 1.80] | 0.8803 |
|  |  |  |  |
| PANAS Positive | 5.63 (2.45) | [0.40, 10.86] | 0.0365 |
|  |  |  |  |
| PANAS Negative | -0.49 (2.93) | [-6.74, 5.76] | 0.8690 |
|  |  |  |  |
| Well-Being | -9.60 (7.30) | [-25.66, 6.47] | 0.2154 |
|  |  |  |  |
| Distress | -3.55 (5.38) | [-15.40, 8.29] | 0.5228 |
|  |  |  |  |
| Distress/Craving Sum (Absolute Difference in Sides) via LVFT | -12.70 (8.57) | [-30.97, 5.57] | 0.1590 |
|  |  |  |  |
| Distress/Craving Sum (Absolute Difference in Sides) via CTHEV | -3.42 (2.66) | [-9.08, 2.25] | 0.2181 |
|  |  |  |  |

[1] Separate models were constructed examining one independent variable at a time. Baseline OCS was based on the prior week score for calculating percent change.

[2] The outcome is scaled as the difference in percent change from baseline. Positive values for the parameter estimate indicates that higher values of the independent variable are associated with more favorable response to sham treatment, while negative values for the parameter estimate indicates that higher values of the independent variable are associated with more favorable response to active treatment.

Abbreviations: - Hamilton Depression Rating Scale; HARS - Hamilton Anxiety Rating Scale; PANAS - Positive and Negative Affect Schedule; LVFT - Lateral Visual Field Test; CTHEV - Computerized Test for Hemispheric Emotional Valence

Table 12. Comparison of HDRS Scores Between Active and Sham Treatment

All Randomized Patients with Complete Data

| **Statistic** | **Active** | **Sham** | **Difference (Active - Sham)** |
| --- | --- | --- | --- |
| n | 16 | 16 | 16 |
| Mean (SD) | 8.8 (10.3) | 13.3 (12.9) | -4.5 (6.7) |
| Median | 4.5 | 8.0 | -3.0 |
| Min, Max | 0.0, 37.0 | 0.0, 46.0 | -17.0, 7.0 |
| 95% CI | (3.3, 14.2) | (6.4, 20.1) | (-8.1, -0.9) |
| p-value |  |  | 0.0087 |

[1] A paired t-test was performed to compare the post-baseline HDRS score on active treatment versus sham treatment (Difference column). A positive value for the difference favors sham treatment, while a negative value favors active treatment.

[2] One-sided lower p-values were calculated for all tests.

Table 13. Comparison of HARS Scores Between Active and Sham Treatment

All Randomized Patients with Complete Data

| **Statistic** | **Active** | **Sham** | **Difference (Active - Sham)** |
| --- | --- | --- | --- |
| n | 16 | 16 | 16 |
| Mean (SD) | 8.0 (13.2) | 14.3 (16.0) | -6.3 (11.4) |
| Median | 2.5 | 4.5 | -2.0 |
| Min, Max | 0.0, 42.0 | 0.0, 54.0 | -29.0, 9.0 |
| 95% CI | (1.0, 15.0) | (5.8, 22.8) | (-12.4, -0.2) |
| p-value |  |  | 0.0214 |

[1] A paired t-test was performed to compare the post-baseline HARS score on active treatment versus sham treatment (Difference column). A positive value for the difference favors sham treatment, while a negative value favors active treatment.

[2] One-sided lower p-values were calculated for all tests.

Table 14. Comparison of PANAS Positive Scores Between Active and Sham Treatment

All Randomized Patients with Complete Data

| **Statistic** | **Active** | **Sham** | **Difference (Active - Sham)** |
| --- | --- | --- | --- |
| n | 17 | 17 | 17 |
| Mean (SD) | 18.5 (3.8) | 17.5 (4.7) | 1.0 (4.1) |
| Median | 18.0 | 17.0 | 1.0 |
| Min, Max | 11.0, 25.0 | 11.0, 25.0 | -9.0, 7.0 |
| 95% CI | (16.6, 20.5) | (15.1, 19.9) | (-1.1, 3.1) |
| p-value |  |  | 0.8330 |

[1] A paired t-test was performed to compare the post-baseline PANAS Positive score on active treatment versus sham treatment (Difference column). A positive value for the difference favors active treatment, while a negative value favors sham treatment.

[2] One-sided lower p-values were calculated for all tests.

Table 15. Comparison of PANAS Negative Scores Between Active and Sham Treatment

All Randomized Patients with Complete Data

| **Statistic** | **Active** | **Sham** | **Difference (Active - Sham)** |
| --- | --- | --- | --- |
| n | 17 | 17 | 17 |
| Mean (SD) | 8.5 (4.5) | 8.8 (6.4) | -0.2 (5.2) |
| Median | 7.0 | 5.0 | 0.0 |
| Min, Max | 5.0, 19.0 | 5.0, 23.0 | -13.0, 11.0 |
| 95% CI | (6.2, 10.8) | (5.5, 12.0) | (-2.9, 2.4) |
| p-value |  |  | 0.4269 |

[1] A paired t-test was performed to compare the post-baseline PANAS Negative score on active treatment versus sham treatment (Difference column). A positive value for the difference favors sham treatment, while a negative value favors active treatment.

[2] One-sided lower p-values were calculated for all tests.

Table 16. Comparison of Well-Being Scores Between Active and Sham Treatment

All Randomized Patients with Complete Data

| **Statistic** | **Active** | **Sham** | **Difference (Active - Sham)** |
| --- | --- | --- | --- |
| n | 16 | 16 | 16 |
| Mean (SD) | 7.1 (2.0) | 6.4 (2.1) | 0.6 (1.9) |
| Median | 7.5 | 7.0 | 0.5 |
| Min, Max | 2.0, 10.0 | 3.0, 9.0 | -3.0, 5.0 |
| 95% CI | (6.0, 8.1) | (5.3, 7.6) | (-0.4, 1.6) |
| p-value |  |  | 0.9008 |

[1] A paired t-test was performed to compare the post-baseline Well-Being score on active treatment versus sham treatment (Difference column). A positive value for the difference favors active treatment, while a negative value favors sham treatment.

[2] One-sided lower p-values were calculated for all tests.

Table 17. Comparison of Distress Scores Between Active and Sham Treatment

All Randomized Patients with Complete Data

| **Statistic** | **Active** | **Sham** | **Difference (Active - Sham)** |
| --- | --- | --- | --- |
| n | 16 | 16 | 16 |
| Mean (SD) | 2.2 (2.7) | 2.4 (2.6) | -0.2 (2.3) |
| Median | 1.5 | 2.0 | 0.0 |
| Min, Max | 0.0, 9.0 | 0.0, 8.0 | -6.0, 5.0 |
| 95% CI | (0.7, 3.6) | (1.0, 3.8) | (-1.4, 1.1) |
| p-value |  |  | 0.3767 |

[1] A paired t-test was performed to compare the post-baseline Distress score on active treatment versus sham treatment (Difference column). A positive value for the difference favors sham treatment, while a negative value favors active treatment.

[2] One-sided lower p-values were calculated for all tests.
